# Update on *Giardia*: Highlights from the seventh International *Giardia* and *Cryptosporidium* Conference

**DOI:** 10.1051/parasite/2020047

**Published:** 2020-08-13

**Authors:** André G. Buret, Simone M. Cacciò, Loïc Favennec, Staffan Svärd

**Affiliations:** 1 Biological Sciences, University of Calgary TN4N1 Calgary (AB) Canada; 2 Department of Infectious Diseases, Istituto Superiore di Sanita 00161 Rome Italy; 3 French National Cryptosporidiosis Reference Center, Rouen University Hospital 1 rue de Germont 76031 Rouen cedex France; 4 EA 7510, UFR Santé, University of Rouen Normandy, Normandy University 22 bd Gambetta 76183 Rouen cedex France; 5 Department of Cell and Molecular Biology, Uppsala University SE 75124 Uppsala Sweden

**Keywords:** Giardia, Giardiasis, Epidemiology, Genomics

## Abstract

Although *Giardia duodenalis* is recognized as one of the leading causes of parasitic human diarrhea in the world, knowledge of the mechanisms of infection is limited, as the pathophysiological consequences of infection remain incompletely elucidated. Similarly, the reason for and consequences of the very specific genome-organization in this parasite with 2 active nuclei is only partially known. Consistent with its tradition, the 7th International *Giardia* and *Cryptosporidium* Conference (IGCC 2019) was held from June 23 to 26, 2019, at the Faculty of Medicine and Pharmacy of the University of Rouen-Normandie, France, to discuss current research perspectives in the field. This renowned event brought together an international delegation of researchers to present and debate recent advances and identify the main research themes and knowledge gaps. The program for this interdisciplinary conference included all aspects of host-parasite relationships, from basic research to applications in human and veterinary medicine, as well as the environmental issues raised by water-borne parasites and their epidemiological consequences. With regard to *Giardia* and giardiasis, the main areas of research for which new findings and the most impressive communications were presented and discussed included: parasite ecology and epidemiology of giardiasis, *Giardia*-host interactions, and cell biology of *Giardia*, genomes and genomic evolution. The high-quality presentations discussed at the Conference noted breakthroughs and identified new opportunities that will inspire researchers and funding agencies to stimulate future research in a “one health” approach to improve basic knowledge and clinical and public health management of zoonotic giardiasis.

## Abstract (fr)

Bien que *Giardia duodenalis* soit reconnu comme l’une des principales causes de diarrhée parasitaire humaine dans le monde, la connaissance des mécanismes de l’infection est limitée, car ses conséquences physiopathologiques restent incomplètement élucidées. De même, la raison et les conséquences de l’organisation génomique très spécifique de ce parasite à deux noyaux actifs ne sont que partiellement connues. Conformément à sa tradition, la 7^ème^ Conférence internationale sur Giardia et Cryptosporidium (IGCC 2019) s’est tenue du 23 au 26 juin 2019, à la Faculté de médecine et de pharmacie de l’Université de Rouen-Normandie, France, pour discuter des perspectives de recherche actuelles dans ce champ. Cet événement de renom a réuni une délégation internationale de chercheurs pour présenter et débattre des avancées récentes et identifier les principaux thèmes de recherche et les lacunes dans les connaissances. Le programme de cette conférence interdisciplinaire comprenait tous les aspects des relations hôtes-parasites, de la recherche fondamentale aux applications en médecine humaine et vétérinaire, ainsi que les problèmes environnementaux soulevés par les parasites d’origine hydrique et leurs conséquences épidémiologiques. En ce qui concerne *Giardia* et la giardiase, les principaux domaines de recherche pour lesquels de nouvelles découvertes et les communications les plus impressionnantes ont été présentées et discutées comprenaient : l’écologie parasitaire et l’épidémiologie de la giardiase, les interactions *Giardia*-hôte, la biologie cellulaire de *Giardia*, les génomes et l’évolution génomique. Les présentations de haute qualité discutées lors de la conférence ont noté des avancées et identifié de nouvelles opportunités qui inspireront les chercheurs et les agences de financement à stimuler la recherche future dans une approche « une seule santé » pour améliorer les connaissances de base et la gestion clinique et de santé publique de la giardiase zoonotique.

The protozoan parasite *Giardia duodenalis* (syn. *G. intestinalis, G. lamblia*) is a leading cause of waterborne and foodborne diarrheal disease worldwide (Ryan et al., p. 153 in [31]). Numerous waterborne outbreaks have been reported globally in the recent years, and it is estimated that over 200 million people are infected annually [22]. This parasite is common in a broad range of mammals, including non-human primates. *Giardia* has been found to pose zoonotic risks, and causes significant losses to the livestock industry ([17]; Polak, p. 157 in [31]; Ongerth, p. 167 in [31]; Sahraoui et al., p. 171 in [31]; Bartley et al., p. 172 in [31]; Scorza et al., p. 176 in [31]; Koester et al., p. 179 in [31]). The infection can cause severe disease, acutely as well as post-infectiously, in the intestine and beyond [19]. In areas of the world with low income and poor sanitation, giardiasis in children may lead to malnutrition, failure to thrive, as well as cognitive impairment ([12, 18]; Garzon et al., p. 203 in [31]; Salimo Muadica et al., p. 207 in [31]). Taking advantage of the recent seventh International *Giardia* and *Cryptosporidium* Conference (IGCC VII) held in Rouen (France; June 23–26, 2019) [31], this article provides an in-depth update on the current understanding of this parasite and the disorders it may cause. The topics will cover the ecology and epidemiology of the parasite, *Giardia* – host interactions, as well as the cell biology, gene expression and genomics of the parasite. The findings presented at the Conference [31] are discussed in the context of the most recent literature in the field to provide a state-of-the-art review.

## Ecology and epidemiology

### *Giardia* in environmental waters, seafood, and fresh produce

*Giardia* causes water- and foodborne outbreaks of diarrheal illness globally [28]. In particular, waterborne transmission is of major importance in the epidemiology of the parasite and still constitutes a serious public health concern [8, 22]. To date, *Giardia* is known to exist in eight distinct assemblages, or genotypes, from A to H, where A and B are infectious to humans.

A few studies on the detection of *Giardia* in water samples were presented at IGCC VII (Cho et al., p. 56 in [31]; Cerever-Aragò et al., p. 129 in [31]; Cirkovic et al., p. 132 in [31]). These studies focused on surface water matrices in Europe (Austria, Serbia) and Korea, and demonstrated a high prevalence of the parasite and the circulation of various assemblages, including assemblages A and B. Another study (Hartdegen et al., p. 197 in [31]) reported the use of loop-mediated isothermal amplification (LAMP) to detect *Giardia* in different matrices.

Foodborne outbreaks of giardiasis have been difficult to document, likely because of limitations in the detection methods [37], but also due to higher vulnerability of the cysts to environmental stress, e.g., desiccation, which may reduce the risk of transmission via food. As fresh produce is among the likely sources of foodborne infection, studies presented at IGCC VII focused on different vegetables, including sprouts, herbs and fruits (Berrouch et al., p. 135 in [31]; Slana et al., p. 137 in [31]). The need for further standardization and cost reduction of the immunomagnetic separation method used to recover *Giardia* cysts from food matrices was underlined at IGCC VII (Ryan et al., p. 154 in [31]).

Edible filtrating molluscs, including oysters, have been proposed as potential sources of human giardiasis [5]. A study presented at IGCC VII showed frequent food contamination with human-infectious assemblage A (Santos et al., p. 57 in [31]). Whether cysts present on food matrices are infectious to humans has not yet been properly assessed.

### Animal epidemiology

The issue of the zoonotic potential of *Giardia* has been a topic of interest for many years, and molecular tools are now widely used to genotype isolates from farm, domestic and wild animals. Several interesting studies were presented at IGCC VII.

A large survey in Scotland (Bartley et al., p. 172 in [31]) used beta-giardin and SSU rRNA markers to show high to moderate prevalence of *Giardia* in cattle, sheep, rabbits, rodents and deer species, and to demonstrate the presence of both zoonotic and host-specific assemblages in all these animal species. A study in Italy (Montalbano di Filippo et al., p. 181 in [31]) compared multi-locus genotypes from animals and humans, and further typed assemblage A isolates with a recently developed, highly polymorphic typing scheme [36], confirming its validity. A study in Algeria (Sahraoui et al., p. 171 in [31]) used two markers to type *Giardia* isolates from lambs and identified assemblage A in a minority of the isolates. Finally, a study of captive non-human primates (Koster et al., p. 179 in [31]) confirmed a high prevalence of giardiasis in these animals, with a predominance of assemblage B [38].

Two studies from Italy focused on wild rodents. The first (Perrucci et al., p. 75 in [31]) reported a high prevalence of *Giardia* in the crested porcupine, but did not include molecular characterization of the isolates. The second (De Liberato et al., p. 76 in [31]) showed the presence of *Giardia microti* in captive voles, reinforcing the concept that wild animals more often harbor host-specific, non-zoonotic species or assemblages [30].

A summary of the clinical importance and public health implications of giardiasis in domestic animals underlined how interpretation of the pathogenic role of *Giardia* is complicated by the presence of multiple infections and complex interactions with the microbiota (Polack and Adjou, p. 157 in [31]). Another study (Scorza et al., p. 176 in [31]) reported a lack of association between assemblages and clinical signs of giardiasis in dogs, and stressed that co-infections and the role of the gut microbiota must be considered.

### Human epidemiology

Differences in the molecular epidemiology of giardiasis at national levels [35] were confirmed at IGCC VII. The majority of the studies pointed to a higher prevalence of assemblage B, in both low-income and high-income settings and in different age groups (Puebla et al., p. 77 in [31]; Muadica et al., p. 106 in [31]; Briones et al., p. 108 in [31]; Srkoicova et al., p. 177 in [31]; Muadica et al., p. 207 in [31]; Koster et al., p. 208 in [31]), with the notable exception of Australia, where assemblage A was more prevalent (Zajaczkowski et al., p. 105 in [31]). Interesting observations emerged from these studies, including the predominance of sub-assemblage BIV in Europe (Koster et al., p. 208 in [31]), the first detection of assemblages C and F in humans in Europe (Srkoicova et al., p. 177 in [31]), and the common occurrence of mixed infections (Puebla et al., p. 77 in [31]; Zajaczkowski et al., p. 105 in [31]). However, these studies differ in the methodologies applied, with some based on one marker, while others used multi-locus genotyping. This makes inter-study comparison difficult and calls for standardization of molecular approaches.

Other epidemiologic investigations, not supported by molecular data, confirmed the high prevalence of giardiasis among children (Garzon, p. 203 in [31]; Latif et al., p. 204 in [31]) and immunosuppressed individuals (Bahadur et al., p. 125 in [31]), and confirmed the risk of association between infection, decreased linear growth and the development of cognitive deficits [1]. Finally, a 14-year survey of giardiasis in Romania (Codrean et al., p. 211 in [31]) showed higher rates of infection in females in urban areas, and demonstrated clear seasonal trends [39].

## *Giardia*-host interactions and disease

*Giardia* was included in the WHO’s Neglected Disease Initiative in 2006, and is now well known to cause significant morbidity in young children [6, 34]. Recent observations suggest that in countries of the World where enteric disease is common, children infected with *Giardia* appear to be protected against diarrheal disease for reasons that remain obscure [9, 43]. In countries with poor sanitation and inadequate water purification systems, individuals may be co-infected with multiple diarrheal disease-causing pathogens [21]. *Giardia* infections may cause significant acute disease, and lead to important post-infectious complications, including post-infectious functional gastrointestinal disorders such as Irritable Bowel Syndrome ([19, 20], Dizdar p. 49 in [31], Koster p. 208 in [31]). In fact, the causal relationship between giardiasis and the development of post-infectious intestinal hypersensitivity has recently been established, and was associated with the loss of intestinal barrier function at the acute stage of the infection [23]. The pathophysiology of the parasite remains incompletely understood, but effects may reach sites remote from active colonization, and can include the joints, the eyes, and the central nervous system [4, 5]. Recent observations, including those presented at the IGCC VII, suggest that the interactions of the parasite with the host, with co-infecting enteropathogens, as well as host diet, play significant roles in the clinical disease outcome (Oliveira p. 45 in [31]; [9, 32]). However, the molecular pathophysiology of giardiasis remains incompletely understood. Similarly, whether re-occurrence of giardiasis after drug treatment in patients may represent reinfection or parasite resistance remains unclear (Fantinatti, p. 109 in [31]). As discussed below, recent developments in “omics” technologies have started to reveal novel pathogenic mechanisms (Svard, p. 16 in [31]).

### Immune responses in giardiasis

*Giardia* does not normally invade healthy mucosal tissues, though recent evidence suggests this may occur under experimental conditions [11]. The infection causes few signs of local inflammation, at least in part because of the potent anti-inflammatory actions of the parasite [10], which appear to be induced by its cysteine proteases [25, 42]. Recent years have seen significant advances in our understanding of innate and adaptive immunity to *Giardia* [41], including in complex animal models such as the Mongolian gerbil (*Meriones unguiculatus*) [33]. Variant-specific surface proteins (VSPs) and their antigenic variation contribute to the chronicity of infection [16]. Overall, *Giardia* appears to activate a combination of Th1, Th2, and Th17 immune responses [24], and recent observations indicate that in mice, the chymase mouse mast-cell Protease-4 regulates intestinal cytokine expression in mature hosts infected with *Giardia duodenalis* [40]. In both humans and animals, infection induces a strong protective adaptive immune response, in which the main player is CD4+ T lymphocyte-mediated production of IgA [16, 33, 41]. Recent findings presented at IGCC VII showed that local elevations in the ratio between Th17 and Treg lymphocytes within the small intestinal lamina propria and Peyer’s Patches increased resistance to infection in mice (Yordanova p. 39 in [31]). In support of this hypothesis, another study visualized increased numbers of IL-17A positive lymphocytes in the Peyer’s Patches and intraepithelial compartments of the small intestine of infected mice (Paerewijck p. 41 in [31]). Interestingly, duodenal mucosal lymphocyte alterations are maintained for many months after the onset of infection in human patients (Dizdar p. 49 in [31]). Consistent with previous observations [2], the presentations also revealed that CD8+ T cells do not contribute to immune protection, but instead, are responsible for the epithelial microvillous damage and disaccharidase impairment observed in giardiasis; infected patients or mice also have elevated serum interferon gamma levels (Singer p. 40 in [31]). These observations coincided with a likely role for macrophages to mediate regulation of the infection, but not to directly control it (Singer p. 40 in [31]). Moreover, *Giardia* was shown to alter macrophage pro-inflammatory signaling by cleaving the p65RelA subunit of the transcription factor Nuclear Factor-Kappa B (Faria p. 62 in [31]). In human intestinal epithelial cells, elegant experiments demonstrated that a gene encoding the RNA-binding protein zinc finger protein 36, tristetraprolin (*TTP*), was implicated in the posttranscriptional production and decay of cytokines via an inhibition of ERK1/2 and P38 phosphorylation (Maayeh p. 42 in [31]). Interestingly, prior infection with *Giardia muris* in mice appears to modulate immune responses to another parasite, *Toxoplasma gondii* (Coelho p. 61 in [31]).

### Production of disease

During the acute phase of infection, *Giardia* actively interacts with the intestinal surface, causing epithelial damage, mucus layer disruptions, and gut microbiota dysbiosis [4, 7, 26, 27, 42]. These effects may occur at the site of infection and beyond, and result at least in part from the actions of *Giardia* excretory and secretory products and the host immune response ([28, 35], Gruettner p. 221 in [31]). *Giardia* cysteine proteases appear to play a critical role in the interactions between host intestinal epithelial cells and the parasite (Peirasmaki p. 89 in [31]; Gruettner p. 221 in [31]), as they do in the microbiota dysbiosis it induces [27]. Elegant studies using murine model systems confirmed that infection with *Giardia* disrupts intestinal microbiota and pave the way towards more research into microbiome-*Giardia* interactions (Starcevich p. 83 in [31]). IGCC VII also shed light on why the onset of clinical signs may be so variable during giardiasis (Buret p. 48 in [31]). In fact, *Giardia* cysteine proteases cleave pro-inflammatory mediators and hence attenuate the pathophysiology of intestinal injury caused by co-infecting attaching-effacing enteropathogens that cause diarrhea via severe inflammation ([21], Buret p. 48 in [31]). The mechanisms implicate activation of beta-defensin and intestinal trefoil factor-3 epithelial anti-microbial peptides, via activation of the NLRP3 inflammasome ([13], Buret p. 48 in [31]). Experiments using the non-mucinogenic human gut cell line Hu-Tu-80 revealed intriguing alterations in mucin MUC2 and MUC5AC mRNA levels when the cells were exposed to *Giardia duodenalis* trophozoites (Tsantarlis p. 84 in [31]), further corroborating the pathogenic effects of this parasite on intestinal mucus physiology [7].

*Giardia* is able to affect host lipid metabolism by altering lipid transporters, which may further contribute to disease (Maertens p. 50 in [31]). *Giardia duodenalis* arginine deaminase is vital to parasite arginine metabolism (Ehret p. 85 in [31]), is polymorphic, and may also represent another family of virulence and immune-evasive factors in this parasite (Klotz p. 79 and p. 88 in [31]). In a cohort of Brazilian children, giardiasis was associated with intestinal epithelial cell damage – shown by elevated intestinal fatty acid binding protein – and elevated plasma IL-17 and IL-10, while plasma IL-8 and IL-5 were decreased (Cascais p. 118 in [31]). Using the human cell line Hu-Tu-80, *Giardia* trophozoites disrupted the epithelial barrier in a hypoxic environment in association with alterations of cellular protein kinases possibly providing a new cell model for such studies (Souza p. 90 in [31]). Also, *Giardia* enolase was shown to contribute to host intestinal epithelial cell damage by causing necroptosis via the apoptosis-inducing factor pathway (Barroeta-Echegaray p. 53 in [31]). Ongoing research is investigating the role of *Giardia* microvesicles in pathogenesis (Midelj p. 66 in [31]). It was then shown that studies into the pathophysiology of epithelial dysfunction will significantly benefit from the use of human small intestinal organoids (Kraft p. 43 in [31], Holthaus p. 80 in [31]). The promotion of post-infectious functional gut disorders by giardiasis has been well established [19, 20], Dizdar p. 49 in [31], Koster p. 208 in [31]). One study presented at IGCC VII demonstrated that patients with symptomatic or sub-clinical infections have elevated serum levels of serotonin, a key mediator of intestinal motility, which is a functional marker of intestinal hypersensitivity in Irritable Bowel Syndrome (Singer p. 40 in [31]).

### Anti-giardial drug research at IGCC

Metronidazole remains the drug of choice in most parts of the world where giardiasis is treated. However, users may have side effects such as vomiting or nausea, and treatment failure, which are more and more frequently reported, and make it imperative to identify and develop new low side effect, and low toxicity antigiardial compounds. Thus, several studies on anti-giardial drugs were presented at the IGCC VII (Leitsch p. 188 in [31]; Gadelha p. 191 in [31]; Hart p. 235 in [31]). *Giardia* NAD+-dependent deacetylases such as sirtuins, have been presented as potential new drug targets, notably for nicotinamide (Lagunas-Rangel p. 189 in [31]). Novel systems biology studies to investigate metronidazole resistance are ongoing (Emory-Corbin p. 236 in [31]). Nitroreductases are a group of *Giardia* molecules of ongoing interest in the research on metronidazole resistance (Krakovka p. 217 in [31]). A variety of therapeutic approaches are topics of ongoing investigations. They include the identification of natural products and probiotics with anti-giardial activities.

## Cell biology, gene expression, and genomics

### Cell biology

*Giardia* has a unique cell biology and it is more and more used as a model system to understand conserved eukaryotic cellular features but also *Giardia*-specific research [14]. Peripheral Vacuoles (PVs) are organelles found in *Giardia* that have been shown to mediate bulk fluid phase uptake, and this is an important point of entry for nutrients [15]. The use of 3D super resolution light microscopy and single molecule localization showed that the PV population is heterogeneous when it comes to morphology and volume (Santos et al., p. 51 in [31]).The PV protein interactome was further dissected and a dynamic binding to phospholipid phosphatidylinositols (PIP) was seen from many different PV proteins containing different PIP-modules (Faso et al., p. 86 in [31]). Manipulation of the levels of the PIP-binding proteins affected the function and morphology of the PVs. This shows that the PVs have many more roles than just being lysosomal-like organelles [44].

*Giardia* trophozoites have four pairs of flagella that undergo a maturation cycle in which certain flagellar pairs are inherited and others are built *de novo* over several generations. Using elegant pulse-chase imaging experiments of labelled beta-tubulin, it was shown that the left caudal is the oldest flagellum, the right caudal flagellum is the third oldest, and the two anterior are the second oldest flagella (Williams et al., p. 87 in [31]). The ventral and posterolateral flagella, as well as the ventral disc and median body, are built *de novo* each generation and post translational modifications of tubulin was suggested to regulate these changes. The ventral disc architecture and composition was studied using CRISPR-interference (CRISPRi)-mediated knockdowns and high-resolution live imaging, electron microscopy, and biophysical assays (Hagen et al., p. 52 in [31]; Hilton and Dawson, p. 82 in [31]). In total 90 disc-associated proteins (DAPs) have been identified and the knock-down of 10 selected DAPs showed defects in disc biogenesis, stability and flexibility, resulting in aberrant disc structures with limited capabilities in attachment.

The mitosomes of *Giardia* are one of the most reduced mitochondria found to date [15]. The only known metabolic pathway is the synthesis of iron-sulphur clusters (ISC pathway) [23]. The identification and characterization of a BolA protein in *Giardia* further defined the ISC pathway (Motyckova et al., p. 222 in [31]). The dynamics of mitosomes in *Giardia* was studied using the FIB-SEM technique (Voleman et al., p. 24 in [31]) and it showed that the *Giardia* mitosomes are actually asymmetrical and that mitosomal division involves association with the endoplasmic reticulum (ER).

### Gene expression

Several studies using different omics techniques to study gene-expression and protein-modification changes during host-parasite-interactions and differentiation were presented at the IGCC VII.

The first high-resolution proteome for *Giardia* across encystation was generated using mass spectrometry, quantifying ~3600 proteins across biological replicates (Balan et al., p. 32 in [31]). This proteome dataset can be used for large-scale transcriptomic-proteomic correlation studies, verifying protein expression, as well as providing insights into parasite transmission. This was complemented by a study using transcriptomic analyses (RNAseq) of gene-expression changes every 3rd hour during encystation, and it suggested a role of epigenetic gene-expression control in encystation (Rojas-Lopez et al., p. 219 in [31]). Biotinylation of cyst-wall proteins and mass spectrometry was used to identify protein-protein interaction partners in encystation-specific vesicles (ESVs) (Vinopalova et al., p. 223 in [31]; Markova et al., p. 228 in [31]). Studies of protein modifications during trophozoite growth and encystation showed that *Giardia* lack the ability to methylate arginine-residues in proteins and only do lysine-methylation (Emery-Corbin et al., p. 31 in [31]).

The cross-talk between parasites and host cells was studied via differential gene expression analyses on the RNA level using RNASeq (transcriptomics) (Svard, p. 16 in [31]). This showed extensive, sequential gene-expression changes in both the parasite (Peirasmaki et al., p. 89 in [31]) and in the host cells (Maayeh et al., p. 42 in [31]) during interactions. It will be very interesting to follow this up in the small intestinal organoid/parasite interaction systems described at the meeting (Kraft et al., p. 43 in [31]; Holthaus et al., p. 80 in [31]).

### Genomes

The first *Giardia* genome (WB isolate, assemblage A1) was published in 2007 [29] and it has since then played an essential role in the progress of *Giardia* research. Genomics in *Giardia* is currently developing fast and this was reflected at the IGCC VII.

*Giardia muris* has been used as a natural infective model of *Giardia* infection for many years. However, very little is known about the genome and biology of this parasite since it is recalcitrant to axenization. A *Giardia muris* draft genome was presented at the meeting (Xu et al., p. 16 in [31]). The *G. muris* genome is the first non-*G. duodenalis* species to be sequenced and it shows high levels of streamlining, amongst the most densely encoded ever described for a nuclear eukaryotic genome. *Giardia muris* and *G. duodenalis* share many known or predicted virulence factors, including cysteine proteases and a large repertoire of cysteine-rich surface proteins involved in antigenic variation. Reconstruction of metabolic pathways from the *G. muris* genome and phylogenetic inferences suggest significant metabolic differences to *G. duodenalis.* Additionally, *G. muris* encodes proteins that might be used to modulate the prokaryotic microbiota.

Using updated and essentially complete versions of the *G. intestinalis* WB, GS and P15 genomes, a detailed comparative study of 47 *G. duodenalis* genomes from isolates grown *in vitro*, belonging to different assemblages (34 A, 12 B and 1 E), has been performed (Jex et al., p. 20 in [31]). This can be used to further explore genetic variation linked to host-specificity, virulence and drug resistance.

New genomic data was presented from sequencing of individual and pooled cysts from the dog assemblages C and D (Kooyman et al., p. 22 in [31]; [23]). All the genomes were nearly complete (>99% single copy genes observed). The phylogenetic distance based on the whole genome between assemblage C and D was about the same as between assemblage A and B, and the assemblage C and D isolates were more closely related to assemblage B than to assemblage A. This showed that whole genome sequencing from a single *Giardia* cyst is possible and makes the genomes of unculturable *Giardia* assemblages accessible for analyses in the future.

The use of genomic information during water-borne outbreaks of *Giardia* was shown in an investigation of an outbreak that occurred in a small village in the north of Italy (Sannella et al., p. 21 in [31]). Cysts were purified from patient samples and the genomes of 6 isolates were sequenced after genome amplification. This showed for the first time that it is useful to perform genomic analyses during *Giardia* outbreaks.

This conference provided an opportunity to underline key issues for future research such as improvement of *Giardia* detection in clinical and environmental samples, as well as food matrices, the actual zoonotic potential of the parasite and clinical importance and public health implications of giardiasis in domestic animals, further development of “omics” technologies to reveal novel pathogenic mechanisms and mechanisms of treatment failures. The high-quality presentations discussed at the conference noted breakthroughs and identified new opportunities that will inspire researchers and funding agencies to stimulate future research in a “one health” approach to improve basic knowledge and clinical and public health management of zoonotic giardiasis.
